# DNA损伤修复系统与肺癌的药物治疗

**DOI:** 10.3779/j.issn.1009-3419.2022.101.24

**Published:** 2022-06-20

**Authors:** 琳琳 张, 凡路 孟, 殿胜 钟

**Affiliations:** 300052 天津，天津医科大学总医院肿瘤科 Department of Medical Oncology, Tianjin Medical University General Hospital, Tianjin 300052, China

**Keywords:** 肺肿瘤, DNA损伤, DNA修复, 抗肿瘤药物, Lung neoplasms, DNA damage, DNA repair, Antineoplastic agents

## Abstract

DNA损伤修复（DNA damage repair, DDR）系统在维持“基因组稳定性”方面发挥重要作用。DNA损伤的累积及DDR功能减弱，都会促进肿瘤发生、发展，同时也给肿瘤的药物治疗提供机会和靶点。在肺癌治疗中，很多抗肿瘤药物发挥作用都与DNA损伤和修复密切相关。从导致DNA损伤的经典化疗药物、新型抗体偶联药物，到抑制DNA修复的靶向药物，以及免疫检查点抑制剂，这些药物在发挥抗肿瘤活性过程中，都直接或间接涉及DNA损伤及修复。本文综述了DNA损伤和修复在上述药物发挥抗肿瘤活性中的作用，并汇总了上述各类药物在肺癌治疗中的应用现状及临床探索，以期通过DNA损伤和修复为切入点，摸索更精准和有效的肺癌治疗策略。

## Abstract (en)

DNA damage repair (DDR) system plays an important role in maintaining of genomic stability. Accumulation of DNA lesions or deficiency of DDR system could drive tumorigenesis as well as promote tumor progression; meanwhile, they could also provide therapeutic opportunities and targets. Of all the antineoplastic agents of lung cancers, many of them targeted or were associated with DNA damage and repair pathways, such as chemotherapies and antibody-drug conjugates which were designed directly causing DNA damages, targeted drugs inhibiting DNA repair pathways, and immune-checkpoint inhibitors. In this review, we described the role of DNA damage and repair pathways in antitumor activity of the above agents, as well as summarized the application and clinical investigations of these antineoplastic agents in lung cancers, in order to provide more information for exploring precision and effective strategies for the treatment of lung cancer based on the mechanism of DNA damage and repair.

人体DNA每天受到上万次的内源性和外源性因素导致的损伤^[1]^。与此同时，体内DNA损伤修复（DNA damage repair, DDR）系统的基因通过修复内源性和外源性因素导致的DNA损伤，在修复基因变异和维持人体细胞“基因组稳定性”方面发挥重要作用。DDR系统主要包含5条重要的修复通路：碱基切除修复（base excision repair, BER）、同源重组（homologous recombination, HR）、错配修复（mismatch repair, MMR）、核苷酸切除修复（nucleotide excision repair, NER）、非同源末端连接（non-homologous end joining, NHEJ）^[2]^。DDR基因的突变或异常，会导致DNA损伤修复功能的缺失，降低了细胞对于内源性和外源性刺激修复能力，促进基因变异的累积，导致肿瘤的发生^[3]^。研究^[4-6]^显示，肺癌的基因组表达谱显示出较高的体细胞变异率，约60%的非小细胞肺癌（non-small cell lung cancer, NSCLC）中存在染色体的多倍体变异，30%-50%的肺癌存在基因组重复的现象，同时，肺癌较其他癌种有较高的肿瘤突变负荷（tumor mutation burden, TMB），这些都与DDR系统异常存在相关性。此外，基因变异率增加也更容易引发肺癌驱动突变的产生，包括表皮生长因子受体（epidermal growth factor receptor, *EGFR*）、*MYC*、*RAS*、*PIK3CA*、间变性淋巴瘤激酶（anaplastic lymphoma kinase, *ALK*）等癌基因激活和/或肿瘤抑制功能的丧失，这些基因改变可直接导致肺癌的发生^[7, 8]^。

DNA损伤和DDR功能异常，除了可导致肿瘤发生，也给肿瘤的治疗提供了机会和靶点^[9]^。在肺癌治疗中，很多抗肿瘤药物发挥作用都与DNA损伤和DNA修复系统密切相关。直接导致DNA损伤的药物，会在短时间内急剧增加肿瘤细胞基因组不稳定性；若肿瘤细胞中损伤的DNA不能被及时、有效地修复，则其会很快死亡^[10]^。靶向抑制DRR系统中关键蛋白的药物，可以利用肿瘤细胞DDR缺陷，通过“合成致死”原理诱导肿瘤细胞死亡^[11, 12]^。此外，DDR异常导致肺癌细胞基因组变异率高还会引起肿瘤免疫微环境的改变，影响免疫检查点抑制剂（immune checkpoint inhibitors, ICIs）的治疗效果^[13]^。可见，上述药物发挥抗肿瘤活性都直接或间接与DDR系统相关。本文就DNA损伤和修复与肺癌药物治疗的关系进行综述，并汇总与DDR相关药物在肺癌治疗中的应用现状及临床探索。

## 1 化疗药物——导致DNA损伤的治疗方式

化疗药物中，拓扑异构酶抑制剂、烷化剂、DNA链交联剂等治疗均可以诱导不同形式的DNA损伤。上述药物以药物原型和抗体偶联药物（antibody-drug conjugates, ADCs）的形式，在NSCLC和小细胞肺癌（small cell lung cancer, SCLC）的治疗中发挥重要作用。

### 1.1 经典化疗药物——直接导致肿瘤细胞DNA损伤

含铂双药化疗一直是肺癌治疗的基石^[14]^。铂类化疗药物抗癌的主要机制是与DNA链上的碱基作用，形成非特异性的铂-DNA交联，改变其正常复制模板的功能，引起DNA损伤。这种损伤影响了DNA的复制、细胞的分裂，导致细胞死亡^[15]^。

拓扑异构酶抑制剂是治疗SCLC最常用的药物之一。拓扑异构酶Ⅰ抑制剂——拓扑替康、伊立替康，被指南推荐用于铂耐药SCLC的治疗。拓扑替康、伊立替康通过与DNA/拓扑异构酶Ⅰ聚合物形成稳定的共价复合物，从而使断裂的DNA单链不能重新接合，阻止DNA复制、抑制RNA合成，为细胞周期S期特异性药物。拓扑异构酶Ⅱ抑制剂——依托泊甙，被指南推荐用于SCLC的一线治疗。依托泊苷是鬼臼毒的半合成衍生物，其干扰DNA拓扑异构酶Ⅱ使DNA断裂重新连接的反应，抑制有丝分裂，使得细胞分裂停止于S期或早G_2_期，诱导细胞凋亡^[16]^。

### 1.2 ADCs——更精准靶向DNA损伤的途径

ADCs是通过化学反应，将重组单克隆抗体与小分子细胞毒性药物通过连接分子结合所形成的新分子。这些ADCs能够通过单克隆抗体识别肿瘤细胞表面“特异性抗原”，通过内吞作用将小分子细胞毒性药物转移入细胞内，发挥精准抗肿瘤活性。目前，小分子细胞毒性药物主要包括导致DNA损伤类药物和抑制微管形成类药物。由于“特异性抗原”在正常细胞表达量少或不表达，因此，ADCs药物赋予小分子细胞毒性药物主动靶向的功能，更精准地靶向肿瘤细胞导致其DNA损伤和细胞死亡^[17]^。

依喜替康（exatecan, DX-8951, DXd）是一种高效拓扑异构酶Ⅰ抑制剂，DXd和来酰亚胺-GGFG链接分子（maleimide-GGFG peptide linker）组成的复合物Deruxtecan，与不同单克隆抗体组合形成不同的ADCs药物。曲妥珠单抗-德鲁替康（Trastuzumab Deruxtecan, T-DXd, DS-8201）是由人源化表皮生长因子受体2（human epidermal growth factor receptor 2, HER2）单克隆抗体和Deruxtecan组成。Ⅱ期DESTINY-Lung01研究在HER2过表达或*HER2*突变的经治NSCLC患者中对T-DXd疗效进行了评估，结果发现，在HER2过表达患者中，患者的疾病客观缓解率（objective response rate, ORR）为24.5%，HER2 immunohistochemistry（IHC）2+和3+的缓解持续时间（duration of response, DOR）分别为5.8个月和6个月，疾病控制率（disease control rate, DCR）分别为66.7%和80%^[18]^；在*HER2*突变的患者中，T-DXd的ORR为55%，中位无疾病进展期（progression free survival, PFS）为8.2个月，中位总生存期（overall survival, OS）为17.8个月^[19]^。德帕瑞妥单抗（Patritumab Deruxtecan, HER3-DXd, U3-1402）是由抗HER3抗体和Deruxtecan组成，在EGFR-酪氨酸激酶抑制剂（tyrosine kinase inhibitors, TKIs）耐药的晚期NSCLC患者中，HER3-DXd治疗的ORR为39%，中位PFS为8.2个月^[20]^。Datopotamab deruxtecan（Dato-DXd, DS-1062a）是由抗TROP2单克隆抗体和Deruxtecan组成，剂量爬坡研究提示在经治NSCLC中，Dato-DXd治疗的ORR为25%（8 mg/kg）、21%（6 mg/kg）、23%（4 mg/kg），中位PFS为5.4个月（8 mg/kg）、8.2个月（6 mg/kg）、4.3个月（4 mg/kg）^[21]^。

SN-38是拓扑异构酶Ⅰ抑制剂伊立替康的活性代谢产物。Sacituzumab Govitecan（IMMU-132）是由抗TROP2单克隆抗体和SN-38组成，在经治NSCLC中IMMU-132的ORR仅为17%，中位PFS为5.2个月，中位OS为9.5个月^[22]^。

多柔比星是一种经典拓扑异构酶Ⅱ抑制剂。SGN-15是由抗Lewis-Y单克隆抗体和多柔比星组成的ADCs药物，在经治的NSCLC中SGN-15联合多西他塞不及多西他塞单药的ORR（6% *vs* 21%），但中位PFS为10.9周 *vs* 7.0周略长于多西他塞单药^[23]^。

Pyrrolobenzodiazepine（PBD） 是一类抗肿瘤抗生素，其二聚体可与肿瘤细胞DNA小沟结合并形交联，阻止DNA与转录因子结合，影响肿瘤DNA复制。Rovalpituzumab tesirine（Rova-T）是由抗δ样蛋白3（Delta-like protein 3, DLL-3）单克隆抗体和PBD组成的ADCs药物。DDL-3是一种抑制性NOTCH配体，在80%的SCLC表面中高表达。Ⅰ期临床试验^[24]^提示Rova-T在DLL-3高表达（> 50%）的经治SCLC患者中的ORR能达到38%，然而在随后的Ⅲ期随机对照TAHOE研究^[25]^中，Rova-T二线治疗广泛期SCLC的OS仅为6.3个月，不及二线标准治疗拓扑替康8.6个月（HR=1.46, 95%CI: 1.17-1.82）。Ⅲ期随机对照MERU研究^[26]^探索了Rova-T对比安慰剂在对一线化疗有响应的DLL-3高表达的广泛期SCLC维持治疗中的疗效，同样，结果也并没有发现Rova-T维持能显著改善患者OS获益（HR=1.07, 95%CI: 0.84-1.36）。另一项Rova-T联合Nivolumab±Ipilimumab用于经治广泛期SCLC患者的Ⅰ期/Ⅱ期研究^[27]^提示Rova-T联合方案在当前的剂量水平和给药方案下耐受性不佳。

## 2 靶向DDR通路的治疗——“合成致死”在肺癌领域的初步探索

靶向药物的出现使驱动基因阳性NSCLC患者的生存率有了很大提高。然而，对于驱动基因阴性NSCLC以及SCLC患者的靶向治疗仍有待探索。近年来，靶向聚ADP核糖聚合酶1（poly ADP-ribose polymerase 1, PARP1）、共济失调毛细血管扩张和Rad3相关蛋白（ataxia telangiectasia mutated and Rad3 related kinase, ATR）、WEE1、细胞周期检查点激酶1（checkpoint kinase 1, Chk1）等DDR通路内蛋白的药物，在肺癌的治疗中也进行了初步探索^[10]^。其中，以PARP1抑制剂探索相对深入。

PARP1是DDR系统中的重要蛋白，参与BER、HNEJ等修复通路的修复和调节。PARP1的作用是通过与单链DNA损伤位点相结合、催化多聚ADP核糖链在蛋白底物上的合成、募集其他DNA修复蛋白到损伤位点，参与DNA损伤修复。PARP1抑制剂通过与PARP1催化位点的结合，导致PARP1蛋白无法从DNA损伤位点上脱落，被束缚在DNA上的PARP1在DNA复制时会导致DNA复制叉停滞，转变成DNA双链损伤（double strand break, DSB）。DSB由HR或NHEJ通路进行修复，HR是高保真的精准修复通路，而NHEJ修复不需要DNA模板容易出现DNA突变及变异。BRCA1/2是HR通路上的关键蛋白。当细胞中存在*BRCA1/2*基因变异以致HR通路修复缺失（HR deficiency, HRD）时，由PARP1抑制剂导致的DSB不能通过HR精准修复，只能通过NHEJ修复，没能被修复的DSB以及由NHEJ修复累积的大量基因变异会导致细胞的死亡，这就是被称为“合成致死”的PARP1抑制剂抗肿瘤作用的机制。具体说来，“合成致死”是指两种不同的基因或蛋白同时发生变化导致细胞死亡的方式，而这两种基因或蛋白中只有一种异常则不会导致细胞死亡。此外，许多肿瘤细胞虽然没有*BRCA1/2*基因变异，但是由于HR通路其他蛋白变异也能导致HRD，致使这种肿瘤细胞也对PARP1抑制剂敏感，这大大扩展了PARP1抑制剂的应用范围^[11, 12]^。

### 2.1 PARP1抑制剂在SCLC治疗中的临床探索

SCLC中普遍存在*TP53*和*RB1*基因的缺失或失活，这些基因的变异会抑制细胞周期停滞、影响DNA损伤的修复，使SCLC细胞出现基因变异率高、基因组不稳定性强等特点；同时，SCLC细胞生长速度快，DNA复制的压力大，对DDR通路也有较强依赖性^[28, 29]^。因此，直接攻击或导致DNA损伤的药物，譬如铂及依托泊甙在SCLC治疗中有较好的疗效；与此同时，抑制DDR的药物也可能在SCLC治疗中起关键作用。研究^[30]^发现，SCLC中存在PARP1蛋白的高表达，因此，推测PARP1抑制剂可能在SCLC的治疗中发挥作用。

广泛期SCLC后线用药是治疗中的难点，PARP1抑制剂在这部分患者中进行过临床探索。他拉唑帕尼（Talazoparib）是一种较强的PARP抑制剂，它不仅可以抑制PARP1，也可以抑制PARP2。在Talazoparib治疗恶性实体肿瘤的Ⅰ期临床研究^[31]^中，共纳入23例SCLC患者（既往治疗线数0线-6线），研究发现，Talazoparib治疗SCLC的ORR为26%，中位PFS为11.1周；其中2例完全缓解（complete response, CR）患者均为铂敏感复发的患者。SUKSES-B研究^[32]^应用PARP1抑制剂奥拉帕利（Olaparib）对铂耐药复发SCLC进行治疗，研究发现，患者ORR仅为6.7%，中位PFS为1.25个月，中位OS为8.56个月。可见，铂敏感状态与后线SCLC PARP抑制剂的治疗效果可能存在相关性。如果在PARP1抑制剂基础上，联合直接或间接导致DNA损伤的药物，在DNA损伤的同时抑制修复，两者联合有可能起到协同增效的作用。利用这种机制，在Olaparib联合替莫唑胺治疗复发SCLC的单臂Ⅰ期/Ⅱ期研究^[33]^中，研究者发现患者ORR能达到41.7%，中位PFS为4.2个月，中位OS为8.5个月。维利帕尼（Veliparib）联合替莫唑胺对比安慰剂联合替莫唑胺治疗复发晚期SCLC的Ⅱ期随机对照研究，发现Veliparib联合替莫唑胺组ORR也能达到39%，对照组仅为14%，但两组中位PFS分别为3.8个月和2.0个月，并没有统计学差异（*P*=0.39）^[34]^。然而，该研究在此基础上进行了SLFN11作为标记物的探索分析，发现在SLFN11表达阳性患者中，Veliparib联合替莫唑胺显著延长患者中位PFS（5.7个月*vs* 3.6个月，*P*=0.009）和OS（12.2个月*vs* 7.5个月，*P*=0.014）^[34]^。SLFN11是HR通路的上游蛋白，抑制HR修复，该研究结果提示，除BRCA1/2蛋白外，寻找合适的HR通路相关蛋白作为标记物，可能是提高PARP1抑制剂治疗SCLC疗效的重要途径。除了PARP1抑制剂与化疗联合，PARP1抑制剂与ICIs的联合在经治SCLC中也进行了探索。MEDIOLA研究应用Olaparib联合Durvalumab对经治（既往治疗线数2线-4线）的SCLC进行探索，结果发现，患者ORR仅为10.5%（并未达到预设的研究终点数35%），中位PFS仅为1.8个月，中位OS为4.1个月；尽管并未取得阳性结果，但是该研究发现对该联合治疗有响应的病例均属于炎症浸润型SCLC^[35]^。Olaparib联合Pembrolizumab对比Pembrolizumab在SCLC二线治疗中进行了探索，结果发现，联合治疗组与单药组的ORR分别为45.5%和10.0%，中位PFS分别为5.93个月和3.53个月（*P*=0.036），中位OS分别为10.43个月和8.34个月（*P*=0.063），转移灶数目、乳酸脱氢酶水平和铂敏感均为影响该联合治疗疗效的独立危险因素^[36]^。此外，Olaparib联合ATR抑制剂（Ceralasertib, AZD6738）在经治的SCLC患者中也进行了探索，结果发现该组合的ORR仅为3.8%，中位PFS为2.75个月（95%CI: 1.77-5.44），中位OS为7.18个月（95%CI: 5.97-10.79）^[32]^。

除了SCLC后线治疗外，PARP1抑制剂在广泛期SCLC一线维持治疗中也进行了探索。Olaparib对比安慰剂在一线对化疗响应的广泛期SCLC患者中进行了维持治疗的研究，发现Olaparib维持组中位PFS为2.3个月对比安慰剂组2.1个月（HR=0.89, 95%CI: 0.51-1.55, *P*=0.68），中位OS分别为9.5个月和14.1个月（HR=1.29, 95%CI: 0.68-2.45, *P*=0.44），均无统计学差异^[37]^。同样，PARP1抑制剂尼拉帕利（Niraparib）对比安慰剂对一线化疗有反应的广泛期SCLC患者也进行了探索，研究发现，Niraparib一线维持组患者的中位PFS为1.54个月对比安慰剂组1.36个月（HR=0.66, 95%CI: 0.46-0.96, *P*=0.024, 2）略有延长，但OS两组分别为9.92个月和11.43个月（HR=1.03, 95%CI: 0.62-1.73, *P*=0.905, 2），没有统计学差异^[38]^。上述临床研究提示PARP1抑制剂在广泛期SCLC一线维持治疗中的疗效并不明显。此外，鲁卡帕利（Rucaparib）联合Nivolumab在一线治疗有效的晚期SCLC的维持治疗中也进行了初步探索，该研究为单臂研究，共纳入20例患者，研究发现联合维持后患者中位PFS为2.67个月（95%CI: 1.87-5.57），中位OS为7.27个月（95%CI: 4.73-9.33）^[39]^。

在广泛期SCLC一线治疗中，PARP1抑制剂联合化疗也进行了探索。ECOG-ACRIN2511研究^[40]^共纳入未经治疗的广泛期SCLC患者128例，研究将患者随机分为Veliparib+依托泊苷+顺铂治疗组和安慰剂+依托泊苷+顺铂治疗组，结果发现，两组ORR分别为46%和42%，中位PFS分别为6.1个月和5.5个月（*P*=0.06），中位OS为10.3个月和8.9个月（*P*=0.17），并未发现统计学差异。该研究结果提示PARP1抑制剂联合化疗并未改善广泛期SCLC一线治疗的疗效。

### 2.2 PARP1抑制剂在NSCLC治疗中的临床探索

驱动基因阴性晚期NSCLC的一线维持治疗一直是研究者们关注的热点领域。近年，化疗药物、抗血管生成药物及ICIs等药物均在晚期NSCLC一线维持治疗中进行过探索并取得阳性结果，那么晚期NSCLC能否获益于PARP1抑制剂的维持治疗更值得期待。2020年美国临床肿瘤学会（American Society of Clinical Oncology, ASCO）会议报道了一项应用Olaparib对比安慰剂在一线对含铂化疗±ICIs治疗有反应的驱动基因阴性晚期NSCLC患者中进行维持治疗的Ⅱ期随机对照研究，该研究共纳入70例患者，结果发现Olaparib维持组中位PFS为16.6周，对比安慰剂组12.0周（HR=0.83, 95%CI: 0.61-1.15, *P*=0.23），中位OS分别为59.4周和31.3周（HR=0.68, 95%CI: 0.37-1.26, *P*=0.22），均无统计学差异^[41]^。Ⅱ期随机对照PIPSeN研究也探索了Olaparib对比安慰剂在对含铂化疗响应的一线晚期NSCLC患者维持治疗中的疗效，研究发现，应用Olaparib维持组患者的中位PFS为2.3个月对比对照组2.1个月（HR=0.89, 95%CI: 0.51-1.55, *P*=0.68），中位OS分别为9.5个月和14.1个月（HR=1.29, 95%CI: 0.68-2.45, *P*=0.44），均无统计学差异；当时由于考虑到ICIs一线维持治疗给晚期驱动基因阴性NSCLC患者带来的PFS甚至OS获益，PIPSeN研究^[42]^在分析了50%纳入患者的生存结果后宣布提前终止。上述研究提示PARP1抑制剂单药维持治疗没有给晚期NSCLC带来生存获益，后面开展的研究正在应用PARP1抑制剂联合ICIs在晚期NSCLC一线维持治疗中进行探索。Ⅲ期随机对照KEYLYNK-006研究，探索Pembrolizumab联合Olaparib对比Pembrolizumab在晚期非鳞NSCLC患者一线维持治疗中的疗效^[43]^；KEYLYNK-008研究^[44]^探索Pembrolizumab联合Olaparib对比Pembrolizumab在晚期鳞状NSCLC患者中一线维持治疗的疗效；ZEAL-1L研究^[45]^探索应用Niraparib联合Pemborlizumab对比Pembrolizumab在晚期NSCLC一线维持治疗中的疗效；目前，上述研究正在进行中。

围绕驱动基因阴性晚期NSCLC患者的一线治疗，PARP1抑制剂为基础的联合治疗模式也进行了探索。一项Ⅲ期随机对照的临床研究^[46]^，纳入970例晚期肺鳞状细胞癌患者，患者被随机分为接受紫杉醇+卡铂+Veliparib组或紫杉醇+卡铂组，共进行6个周期的治疗，主要研究终点为OS。结果发现联合治疗组的OS为11.9个月对比化疗组的11.1个月（HR=0.905, 95%CI: 0.744-1.101, *P*=0.266），中位PFS两组均为5.6个月（HR=0.897, 95%CI: 0.779-1.032, *P*=0.107），ORR两组均为37%，均无统计学差异。该研究显示，一线应用Veliparib联合化疗并不能改善晚期肺鳞癌患者的生存获益。然而，该研究探索性地分析了肺癌52基因的基因检测panel（lung panel 52, LP52）作为生物标记物在预测Veliparib联合化疗中的作用，LP52阳性被定义为检测标本中具有非腺癌的基因特征。结果发现，在LP52阳性的NSCLC患者中，Veliparib联合化疗可延长患者中位OS（14.0个月*vs* 9.6个月，HR=0.66，95%CI：0.49-0.89），而LP52阴性的NSCLC患者中，两组中位OS分别为11.0个月*vs* 14.4个月（HR=1.33, 95%CI: 0.95-1.86），无统计学差异，提示PARP1抑制剂联合化疗一线治疗晚期NSCLC可能在某些特定亚组人群中有更好的治疗效果。此外，JEASPER研究^[47]^在晚期NSCLC患者中探索了一线应用PARP1抑制剂联合ICIs的治疗效果，该研究按照程序性死亡因子配体-1（programmed death-ligand 1, PD-L1）肿瘤细胞阳性比例分数（tumor proportion score, TPS）评分≥50%和1%-49%，将患者分成两个队列，均应用Pembrolizumab联合Niraparib进行治疗。结果发现在PD-L1 TPS评分≥50%患者中ORR为56.3%，DOR为19.7个月，中位PFS为8.4个月，中位OS时间尚未达到；PD-L1 TPS评分在1%-49%患者ORR为20.0%，DOR为9.4个月，中位PFS为4.2个月，中位OS为7.7个月。然而，相比晚期NSCLC一线单独应用Pembrolizumab的生存结果，我们似乎并没有发现Niraparib联合Pembrolizumab治疗能给患者带来更长的生存获益。在KEYNOTE 042研究^[48]^中，Pembrolizumab单药治疗组，PD-L1 TPS评分≥50%患者的DOR为22.0个月，中位PFS为6.5个月，中位OS为20.0个月；PD-L1 TPS评分在1%-49%患者的DOR为17.4个月，中位PFS为4.2个月，中位OS为13.4个月。

驱动基因阳性的晚期NSCLC中，EGFR-TKIs已成为其一线标准治疗，同时，以EGFR-TKIs为基础的联合治疗策略也在不断被探索。Ib期/Ⅱ期随机对照GOAL研究^[49]^探索了吉非替尼联合Olaparib对比吉非替尼在未经治疗的*EGFR*突变晚期NSCLC患者中的疗效，结果发现，联合治疗并没有显著延长*EGFR*突变NSCLC患者的PFS（12.8个月*vs* 10.9个月，HR=1.38，95%CI：1.00-1.92，*P*=0.124）；但亚组分析^[50]^发现，*BRCA1*基因mRNA高表达的患者，联合治疗组的中位PFS为12.9个月对比吉非替尼单药组9.1个月（*P*=0.044, 9），提示伴有BRCA1 mRNA高表达的患者可能从吉非替尼联合Olaparib治疗中获益。此外，还有研究^[51]^发现PARP1介导的自噬与EGFR-TKIs获得性耐药相关，Olaparib可以通过抑制PARP1在埃克替尼耐药的NSCLC细胞中发挥作用。

## 3 免疫治疗——靶向DDR参与改善肿瘤免疫微环境

以程序性死亡因子-1（programmed death-1, PD-1）/ PD-L1为靶点的ICIs的出现改变了NSCLC和SCLC的治疗格局。PD-L1表达、TMB也成为NSCLC ICIs疗效的预测指标。研究^[52]^表明，DDR通路突变患者TMB平均水平较无突变患者显著增高，提示DDR突变可能成为预测ICIs疗效的新标记物。的确，研究^[53]^发现驱动基因阴性晚期NSCLC患者中，DDR基因突变患者较DDR基因野生型患者接受ICIs治疗的ORR更高（30.3% *vs* 17.2%, *P*=0.01）、生存时间更长（中位PFS为5.4个月*vs* 2.2个月，HR=0.58，95%CI：0.45-0.76，*P* < 0.001；中位OS为18.8个月*vs* 9.9个月，HR=0.57，95%CI：0.42-0.77，*P* < 0.001）。在所有DDR通路中，MMR修复通路功能缺陷（mismatch repair deficient, dMMR）引起微卫星不稳定性增高，已被证实与ICIs的疗效相关，并已被美国食品药品监督管理局（Food and Drug Administration, FDA）批准用于泛瘤种预测ICIs疗效的指标^[54]^，其他DDR通路与ICIs疗效的相关性也在探索中。

ICIs并不直接靶向DNA损伤及修复，但是DNA损伤增加、修复异常会通过导致“基因组不稳定性”增强、改变肿瘤免疫微环境，影响ICIs治疗的疗效^[13]^。具体机制可能有：①DDR异常会导致更多的DNA突变事件的发生，这些DNA上的新生突变会编码肿瘤特异性新生抗原，表达在肿瘤细胞表面，诱导CD8^+^ T细胞浸润、识别新生抗原并启动免疫监视反应^[55]^；②累积的DNA损伤还会导致胞浆DNA水平增加，胞浆DNA进而结合环GMP-AMP合成酶（cyclic GMP-AMP synthase, cGAS）并通过STING通路启动T细胞免疫应答^[56]^；③化疗药物，譬如铂类，可以在导致DNA损伤的同时，诱导细胞表面PD-L1表达增高^[57]^。从这些角度上看，ICIs联合化疗或ICIs联合DDR通路抑制剂可能能够改善ICIs的治疗效果。

ICIs联合化疗的治疗模式，在NSCLC和SCLC中均进行过探索。Ⅲ期随机对照KEYNOTE189和KEYNOTE407分别探索了Pembrolizumab联合化疗对比化疗一线治疗非鳞和鳞状NSCLC的疗效，结果发现ICIs联合化疗显著延长患者中位PFS及OS^[58, 59]^。类似的结果在广泛期SCLC一线治疗中也能发现，Ⅲ期随机对照IMPOWER133、CASPIAN研究分别探索了Atezolizumab联合化疗、Durvalumab联合化疗对比化疗在广泛期SCLC一线治疗中的疗效，结果发现，联合组均能显著延长患者中位OS时间^[60, 61]^。

ICIs联合DDR抑制剂的治疗模式，在肺癌中也进行过初步探索，但是大多数研究目前均在进行中。在SCLC中，MEDIOLA研究（Olaparib联合Durvalumab对复发SCLC治疗）以及Olaparib联合Pembrolizumab在二线SCLC中的研究，提示该联合方式在不同联合药物选择、治疗线数以及亚组人群选择中需要进一步探索^[35, 36]^。在NSCLC中，KEYLYNK-006、KEYLYNK-008、ZEAL-1L研究均探索了PARP1抑制剂联合Pembrolizumab在一线维持治疗中的疗效^[43-45]^；此外，在ICIs耐药的NSCLC患者中开展的Olaparib或ATR抑制剂（Ceralasertib, AZD6738）联合Durvalumab的研究也在进行中（NCT03334617）^[62]^。

## 4 总结与展望

在肺癌治疗中，很多抗肿瘤药物发挥作用都与DNA损伤及修复密切相关。经典化疗用药，譬如铂类和拓扑异构酶抑制剂，都是影响DNA复制、直接导致DNA损伤的药物；新型ADCs药物，也是利用细胞毒性药物直接导致DNA损伤，并通过连接的重组单克隆抗体识别肿瘤细胞表面“特异性抗原”，更精准地靶向肿瘤细胞进行杀伤；抑制DDR的药物，譬如靶向DDR通路关键蛋白的PARP1抑制剂，通过“合成致死”的机制杀死肿瘤细胞；此外，DDR异常还会引起肿瘤免疫微环境的改变，直接影响ICIs治疗效果。上述药物在提高肺癌患者治疗疗效、改善患者生存的过程中起到了积极的作用。在此基础上，利用协同增效的机制，上述药物也在肺癌中进行了联合治疗的探索，其中非常引人关注的是新型联合治疗模式“PARP1抑制剂联合化疗”以及“PARP1抑制剂联合ICIs”的疗效。然而，在目前已公布结果的研究中，除了PARP1抑制剂联合替莫唑胺能提高SCLC后线治疗的ORR外，我们并没有发现“PARP1抑制剂联合化疗”、“PARP1抑制剂联合ICIs”在晚期SCLC的一线和一线维持治疗中显著改善患者疗效，也没有发现这些联合在晚期NSCLC一线治疗中给患者带来生存获益。因此，探寻PARP1抑制剂及PARP1抑制剂为基础的联合在肺癌治疗中获益或未获益的可能机制显得尤为重要。首先，肺癌中大部分与PARP1抑制剂相关的临床研究都是在“未经选择”的患者中进行的。精准治疗时代，寻找对PARP1抑制剂有效的优势人群是提高治疗疗效的关键。譬如，SLFN11表达的检测有助于筛选SCLC中PARP1抑制剂治疗的优势人群^[63]^；LP52阳性的驱动基因阴性NSCLC一线应用Veliparib联合化疗有更好的OS结果^[46]^；伴有BRCA1 mRNA高表达的*EGFR*突变的晚期NSCLC应用吉非替尼联合Olaparib比单药吉非替尼有更好的PFS获益^[50]^；此外，也有研究提示铂敏感、乳酸脱氢酶等指标也可能是PARP1抑制剂治疗SCLC获益的独立危险因素^[31, 32, 36]^。其次，联合治疗的药物选择、治疗线数，也有可能是影响疗效和生存结果的重要因素。譬如，Olaparib联合Durvalumab治疗经治（既往治疗线数2线-4线）复发SCLC患者的ORR为10.5%、中位PFS仅为1.8个月，而Olaparib联合Pembrolizumab对经治（2线）SCLC治疗的ORR能达到45.5%，中位PFS为5.93个月^[35, 36]^，提示Olaparib联合不同的ICIs可能产生不同的临床疗效，且治疗线数靠前有可能有更好的治疗效果。由此可见，今后的研究可能需要在优势人群选择、不同药物联合等方面进行深入探索，为提高肺癌治疗的有效率和患者生存提供更为精准和有效的手段和途径。
